# The Effect of the Season on the Time Dependent Changes in Colostrum Lactoferrin Level in Murciano–Granadina Goats in Intensive System Farming

**DOI:** 10.3390/ani14172580

**Published:** 2024-09-05

**Authors:** Mónica Marcela Segura, Silvia Martínez-Miró, Miguel José López, Josefa Madrid, Verónica González, Fuensanta Hernández

**Affiliations:** 1 Department of Animal Production, Faculty of Veterinary, University of Murcia, Campus de Espinardo, 30100 Murcia, Spain; monicamarcela.segurar@um.es (M.M.S.); silviamm@um.es (S.M.-M.); mjlopeza@um.es (M.J.L.); alimen@um.es (J.M.); 2 Universidad Nacional de Colombia, sede Medel-lín, Departamento de producción animal, Facultad de Ciencias Agrarias, Grupo de Investigación en Biodiversidad y Genética Molecular (BIOGEM), Carrera 65 #59A-110, Postal code 050034 Medellín, Colombia; vgonzal@unal.edu.co

**Keywords:** goat colostrum, lactoferrin, immunoglobulin G, Brix refractometer

## Abstract

**Simple Summary:**

This study analyzed changes in the lactoferrin content of Murciano–Granadina goat colostrum in the first 96 h postpartum as a function of parity season. In addition, we evaluated production and composition (chemical and immunological), and the usefulness of a Brix refractometer for estimating the IgG content of colostrum. Lactoferrin is a protein present in colostrum that has antimicrobial properties, and its colostrum content was heavily affected by time since delivery, as was that of the other chemical and immunological components, except fat. The season influenced milk yield and the contents of lactoferrin, immunoglobulin G, fat, protein, and somatic cells. Lactoferrin contents were significantly lower in the winter season. The quality of goat colostrum estimated using the Brix refractometer vs. ELISA method evidenced a strong correlation. Our results confirm the critical role of colostrum feeding within the first postpartum day to the neonates, attributed to its superior quality, particularly for its contents in bioactive proteins, such as lactoferrin and immunoglobulin. Additionally, we substantiate the efficacy and cost-effectiveness of the Brix method as a rapid assay for evaluating colostrum quality, thus validating its practical utility.

**Abstract:**

The aim of this research was to evaluate the effects of postpartum day and parity season on the lactoferrin (LF), immunoglobulin G (IgG), and chemical composition of Murciano–Granadina goat colostrum during the first 96 h after kidding, and the use of the Brix refractometer to estimate IgG content. A herd of 3500 intensively managed Murciano–Granadina dairy goats (45–50 kg body weight) was used. Colostrum samples were collected from days 1 to 4 postpartum in the winter, spring, summer, and autumn. The colostrum composition was assessed using an automated infrared method; the LF and IgG concentrations were measured using an ELISA, and for the Brix percentage, we used a digital refractometer. Colostrum taken on the first postpartum day showed the highest concentrations of LF, IgG, proteins and non-fat solids (NFSs). As the postpartum days progressed, a rapid decrease in the LF, IgG, protein, and NFS contents and the Brix value was observed. In contrast, the lactose content increased steadily until the fourth postpartum day (*p* < 0.001). The season influenced milk yield, LF, IgG, protein, fat, and somatic cell content (*p* < 0.05). LF contents were significantly higher in the spring season, IgG contents were higher in autumn colostrum, and fat components were higher in the winter season. The colostrum Brix value showed a positive correlation with the ELISA colostrum LF (r = 0.716, *p* < 0.001) and IgG (r = 0.894, *p* < 0.001) determination; a 20 mg IgG/mL colostrum concentration corresponded to 18 °Brix. Our results corroborate the importance of feeding colostrum to newborns on the first day after birth, not only because of its high level of IgG but also because of its greater presence of the other bioactive protein compounds such as lactoferrin.

## 1. Introduction

As the syndesmochorial placental structure of goats does not allow for the passage of large molecules from dam to offspring in intrauterine life, goat kids are born hypogammaglobulinemic [1]; therefore, the initial nutrient supply for newborn kids is provided by colostrum, the first secretion from the mammary gland after parturition. Colostrum is rich in the proteins, vitamins, minerals, enzymes, and growth factors necessary for the development and survival of newborn kids. It also provides immunoglobulins (IgG, IgA, and IgM), which protect newborns from infectious agents [2]. Thus, the neonatal acquisition of passive immunity depends on the sufficient consumption of colostral immunoglobulin to protect it against infections during early life. The neonate needs to ingest colostral IgG before the intestine is no longer able to absorb macromolecules and before the immune system is able to generate it independently [3]. One of the important factors in establishing high concentrations of serum IgG in newborn goat kids is the IgG colostrum content; however, many factors can affect the IgG’s availability and absorption, including the time elapsed since delivery, the age of the dam, and the method and volume of colostrum administration [4].

Another important protein found in dairy products is lactoferrin (LF). LF is involved in a range of physiological functions, such as innate immunity, showing antibacterial, antifungal, antiviral, antiparasitic, and anticancer activities. Lactoferrin has the ability to chelate iron or bind to the bacterial surface, giving it antibacterial properties, thus making it a key protein in the defense systems of the mammary gland [5]. High levels of LF in milk will improve its quality, especially its microbiological quality and value as a functional food [6]. LF, first discovered as an iron-binding protein in bovine milk, is produced by epithelial cells and neutrophils and is found in most external mammal secretions, such as milk, reproductive-tract secretions, synovial fluid; and lachrymal and salivary secretions [7]. It has also been shown that administering LC to kids improves their growth and blood parameters due to its immunomodulatory properties [8]. However, the evolution of the LF content in dairy-goat colostrum in the first days postpartum is not well known [9].

Therefore, colostrum intake is crucial for the survival of kids, and its bioactive molecule content decreases rapidly from kidding. Thus, colostrum changes with time to become mature milk, and its composition varies according to factors such as milk yield, diet, breed, season, animal health status, lactation number, body mass, and length of dry period, amongst others [10,11,12]. The colostrum IgG content is considered a quality indicator, and its concentration should be measured before the colostrum is fed to animals in order to reduce the failure of passive-transfer cases, as well as to ensure good health and growth in ruminant neonates in modern dairy farms [13]. The measurement of total proteins in the colostrum provides an indirect measure of IgG concentration and can be performed using refractometry. However, there are some limitations and potential sources of inaccuracies in this method, such as instrument variability and quality, calibration errors, limited measurement range, operator error, temperature sensitivity, interferences with non-IgG solids (such as fat) or contamination, variability in colostrum composition, etc. Therefore, control of all of these variation factors is essential to minimize inaccuracies [14,15]. The objective of this study was to evaluate the effects of postpartum day and season on the chemical composition and immunoglobulin G and lactoferrin contents of Murciano–Granadina goat colostrum in the first 96 h after kidding. In addition, we aimed to assess the Brix refractometer method as a quick and inexpensive analytical tool with which to calculate the IgG levels in goat colostrum.

## 2. Materials and Methods

### 2.1. Animals and Samples Collection

A herd of 3500 intensively managed Murciano–Granadina dairy goats (45–50 kg body weight) located in Mula (Murcia, Spain) was used for this study. The goats were 1–5 years old (replacement rate 20%), with an average of 1.4 and 2.1 kids/litter for primiparous and multiparous goats, respectively. Average herd production per lactation was 250–300 kg in primiparous at 5–7 months, and 550–650 kg in multiparous at 9–10 months. The animals were in good health and were dried off about 12 weeks before parturition. The goats were housed freely in stalls and fed mixed rations twice daily (at 08:00 and 16:00 h). The pre-partum (1.5 kg of dry matter (DM)/day) and lactation basal (2.5 kg of DM/day) diets were formulated to meet the requirements of dairy goats outlined by the NRC [16] (National Research Council, Committee on the Nutrient Requirements of Small Ruminants, Board on Agriculture, Division on Earth, 2007) using the following: corn silage, barley straw, barley, corn, oat, soybean meal, sunflower seed, field pea, beans, sugar beet pulp, malted barley, wheat bran, calcium carbonate, and a vitamin–mineral corrector. The pre-partum diets were supplemented with mono-propylene glycol USP (0.05 mL per head per day) to limit the onset of ketosis and to help prevent pregnancy toxemia [17].

The goat herd was spread across 7 farrowing lots (500 goats per lot) throughout the year and was maintained under natural environmental conditions (temperature and photoperiod). Measurements were taken in four seasons per year, using farrowing plots corresponding with January (average temperature (T): 9.6 °C; average relative humidity (RH): 65%), April (T: 15.2 °C; RH: 53%), June (T: 22.4 °C; RH: 52%), and October (T: 17.3 °C; RH: 64%). Colostrum samples were collected from the first four days of milking postpartum at 24 h intervals, corresponding to 1–4 postpartum days (D1, D2, D3, and D4).

Since lactation number could influence most colostrum components [10], only multiparous goats were used. Goat colostrum samples were collected from 5 batches of 50 goats through mechanical milking in a tank separate from the milk tank, consisting of five replications for each season and day. A total of 80 pooled colostrum samples were collected, including five replicates for each season and day.

Pooled colostrum samples (approx. 2 L) were transferred to the laboratory on ice. Then, several 2 mL aliquots were frozen and stored at −20 °C until we were able to determine the immunoglobulin and lactoferrin concentrations, as well as the Brix values. In addition, we collected aliquots in 40 mL bottles, preserved with azidiol (4 µL/mL), and immediately stored them at 4 °C for chemical composition analyses to determine the colostrum components. We also recorded the kidding dates and milking colostrum yields daily.

### 2.2. Laboratory Analysis

The concentrations of fat, protein, lactose, and non-fat solids (NFSs) and the somatic cell counts (SCCs) of colostrum samples were assessed using an automated infrared method, validated for goat measurement, using a MilkoScan FT6000 analyzer, which consisted of a Fossomatic 6000 somatic cell counter (Foss Electric, Hillerød, Denmark).

The quantitative determination of IgG in colostrum was performed using a specific ELISA quantification kit (Bethyl Laboratories, Inc., Montgomery, TX, USA; reference E50-104) by following the manufacturer’s instructions. The absorbance at 450 nm was measured using a microplate reader (Infinite M200PRO, Tecan Trading AG, Männedorf, Switzerland). For LF analysis, colostrum samples were then centrifuged at 17,800× *g* for 30 min at 4 °C to separate the milk fat. The goat LF concentration was measured in the skim colostrum samples using a specific goat lactoferrin ELISA quantification kit (MyBioSource Inc., San Diego, CA, USA). The procedures were performed according to the manufacturer’s instructions. The absorbance at 450 nm was measured using a microplate reader (Infinite M200PRO, Tecan Trading AG, Männedorf, Switzerland).

The total soluble solids (°Bx) content in colostrum samples was determined using a digital refractometer, Atago 3810 PAL-1 (Atago, Tokyo, Japan), following the manufacturer’s instructions. The refractometer was equipped with a Brix scale ranging from 0 to 53, with an accuracy of ±0.2% at 20 °C. The refractometer allowed for automatic temperature compensation to ensure that accurate measurements were taken without recalibrating at different ambient temperatures. The refractometers were cleaned and calibrated with distilled water at room temperature before each analysis. The °Bx % was recorded 3 consecutive times by transferring 0.3 mL into the device reading well.

### 2.3. Statistical Analysis

SPSS Statistics 28.0 software (IBM SPSS, Chicago, IL, USA) was used, and the data were checked for normal distribution using the Shapiro–Wilk test. A General Linear Model (GLM) analysis was used to determine the effect of postpartum time (days 1, 2, 3, and 4) and season (winter, spring, autumn, and summer), and their interactions on the controlled parameters. In addition, orthogonal contrasts were used for postpartum time. When the effects evaluated were significant, the differences between means were fitted using Tukey’s test. The correlations between variables from different measurements were estimated using Pearson’s correlation coefficients. For all the analyses, *p* < 0.05 was determined to be significant.

## 3. Results

### 3.1. Colostrum Yield

**Figure 1:**
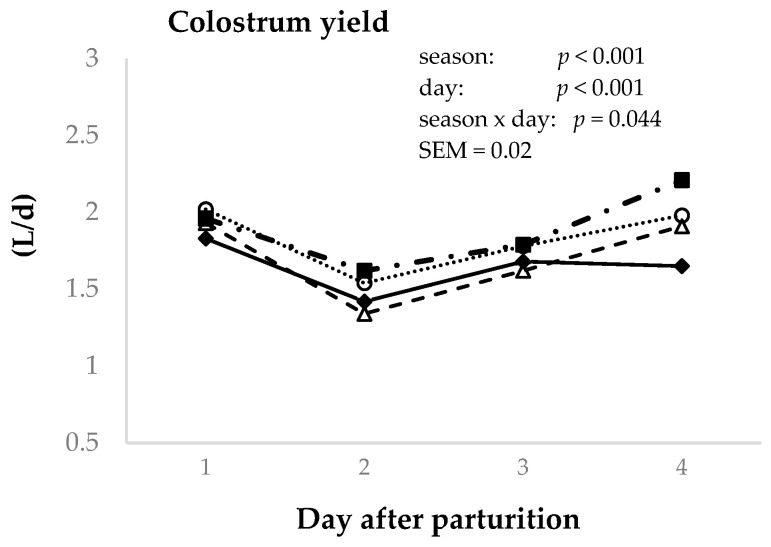
Effect of day postpartum (1st–4th) and season (winter (♦), spring (Δ), summer (■), and autumn (o)) on the colostrum production in Murciano–Granadina goat.

The effects of season and postpartum days on colostrum production are shown in Figure 1. The results showed a season effect (*p* < 0.001), with the highest values being found in summer and autumn (1.9 and 1.8 L/d, respectively) and the lowest in spring and winter (1.7 and 1.6 L/d, respectively); however, autumn colostrum production was not different to that in spring. In addition, a postpartum day effect was also observed (*p* < 0.001), and the highest colostrum yields were recorded on the first and fourth milking days (1.9 L/d for both days), followed by the third and, finally, the second day (1.5 and 1.7 L/d, respectively). Statistical interactions between the season and time of sample collection were found with respect to colostrum yield (*p* < 0.05) in the samples analyzed.

### 3.2. Concentrations of Fat, Protein, Lactose, NFS, and SCC in Goat Colostrum

**Figure 2:**
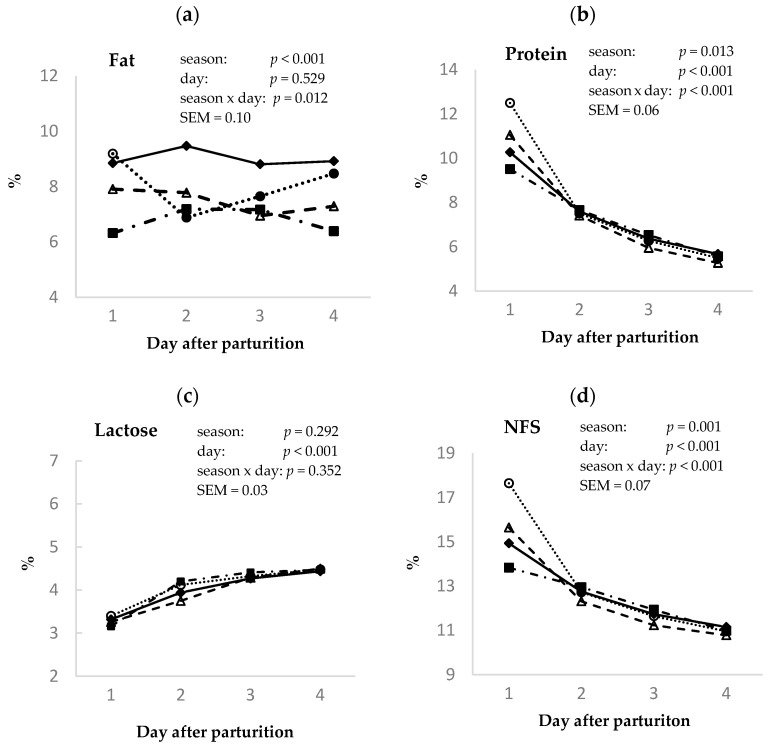
Effect of day postpartum (1st–4th) and season (winter (♦), spring (Δ), summer (■), and autumn (o)) on the colostrum concentration of fat (**a**), protein (**b**), lactose (**c**), and non-fat solids (NFSs) (**d**) in Murciano–Granadina goat.

The sample means for fat, protein, lactose, and non-fat solids (NFSs) in Murciano–Granadina goat colostrum for the first four postpartum milkings and during the four seasons tested are presented in Figure 2.

The fat percentage of colostrum secretions in the first 96 h postpartum (Figure 2a) was affected by season *(p* < 0.001); however, no effect of postpartum time (*p* ≥ 0.05) was detected in the samples analyzed. The highest concentration of fat in colostrum was observed in the winter season, with a mean value of 9.0%, followed by those in autumn, spring, and summer, with means values of 8.0, 7.5, and 6.8%, respectively. An interaction (*p* < 0.05) between the two factors studied was also found for the colostrum fat content.

The total protein concentration in colostrum (Figure 2b) was affected by season (*p <* 0.05). Thus, its content from birth to 96 h postpartum was higher in autumn than in spring and summer but was not different from that in winter (7.9, 7.8, 7.4, and 7.3% for autumn, winter, spring, and summer, respectively). In addition, a linear decrease (*p <* 0.001) in colostrum protein content with increasing days postpartum was found. Thus, the colostrum protein contents were 10.6, 7.5, 6.3, and 5.5% for the first, second, third, and fourth milkings, respectively. In addition, a season × postpartum day interaction (*p* < 0.001) was noticed with regard to the colostrum protein content.

The evolution of lactose content in goat colostrum from birth to 96 h postpartum in the different seasons is shown in Figure 2c. The percentage of lactose was not influenced by seasonal changes (*p* ≥ 0.05). As the days post parturition increased, the lactose contents in colostrum increased linearly (*p* < 0.001), being higher at the third and fourth than first and second milkings (3.3, 4.0, 4.3, and 4.5% for first, second, third, and fourth milking, respectively).

Figure 2d shows how the total NFS colostrum content was affected by the time postpartum and season (*p* < 0.001). The NFS values were the lowest in summer and spring (12.4 and 12.5%, respectively), and they reached their maximum levels in autumn (13.2%), while winter showed intermediate values (12.9%). The total NFS concentration was highest at the first postpartum milking, decreasing linearly (*p* < 0.001) until the fourth controlled day (15.3, 12.7, 11.6, and 11.0% for first, second, third, and fourth milkings, respectively). A season × postpartum day interaction was also observed for the colostrum NFS content (*p* < 0.001).

**Figure 3:**
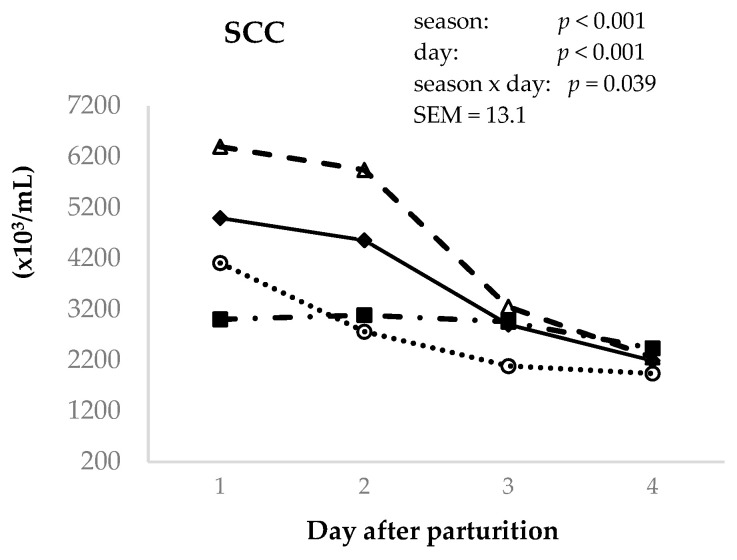
Effect of day postpartum (1st–4th) and season (winter (♦), spring (Δ), summer (■), and autumn (o)) on the colostrum concentration of somatic cell count (SCC) in Murciano–Granadina goats.

The somatic cell count (SCC) of the goat colostrum is displayed in Figure 3. Season (*p* < 0.001) and postpartum time (*p* < 0.001) were observed to affect SCC. For seasons, spring and winter showed the highest counts (4462 and 3796 × 10^3^ cell/mL), and autumn and summer the lowest (2722 and 2870 × 10^3^ cell/mL); however, the SCC levels in summer were the same as those in winter. Regarding the effect of postpartum time, the highest SCC levels were observed in colostrum samples from the first and second milkings. A season x postpartum day interaction was also observed for SCC content (*p* < 0.05).

### 3.3. Immunoglobulin G and Lactoferrin Concentrations

**Figure 4:**
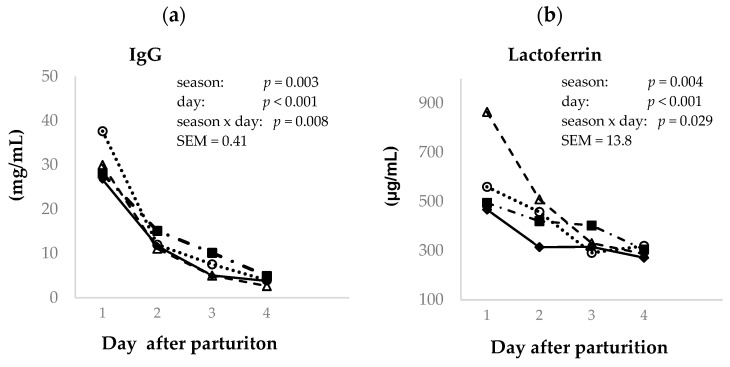
Effect of day postpartum (1st–4th) and season (winter (♦), spring (Δ), summer (■), and autumn (o)) on the colostrum immunoglobulin G (IgG) (**a**) and lactoferrin (**b**) concentration in Murciano–Granadina goats.

Figure 4a shows the evolution of total IgG content in goat colostrum from birth until 96 h postpartum and during the seasons analyzed. Although the GLM analysis indicated a seasonal effect on colostral IgG concentration (*p* < 0.01), when the mean test was performed, no significant differences according to the Tukey test were observed between the means (12.9, 12.4, 13.3, and 15.3 mg IgG/mL for winter, spring, summer, and autumn, respectively). The IgG concentration in colostrum was highest at the first milking (29.5 mg/mL), decreasing linearly (*p* < 0.001) in the second (12.4 mg/mL), third (6.5 mg/mL), and fourth milkings (3.8 mg/mL).

The season was observed to affect the LF colostrum concentration (*p* < 0.01). As is reported in Figure 4b, the highest mean colostrum LF concentration was found in the spring samples (464 µg/mL), and the lowest was observed in the winter samples (342 µg/mL), while those in summer (393 µg/mL) and autumn samples (397 µg/mL) were similar. The colostrum LF concentration during the first 96 h postpartum showed a linear decreasing pattern (*p* < 0.001), similar to the IgG and protein contents. The highest LF concentration was observed on the first day postpartum (582 µg/mL) and in the second (425 µg/mL) and third (334 µg/mL) milkings, and the lowest LF concentration was recorded for the fourth milking (295 µg/mL); however, this was not different to that recorded in the third milking. Interactions (*p* < 0.05) between season and sampling time in relation to the goat colostrum IgG and LF concentrations were detected.

### 3.4. Refractometer

**Figure 5:**
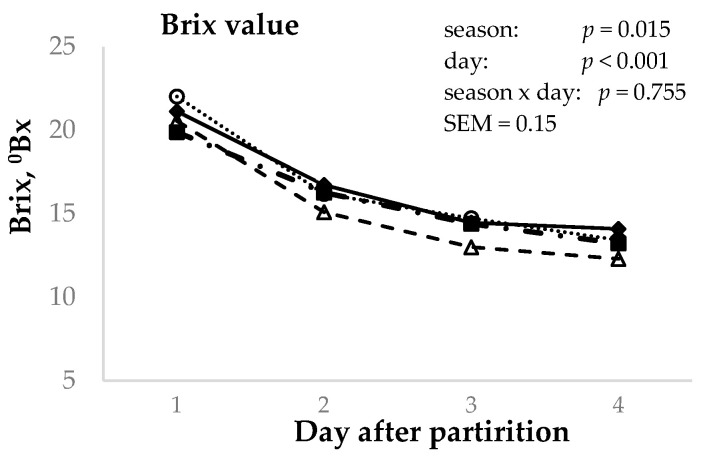
Effect of day postpartum (1st–4th) and season (winter (♦), spring (Δ), summer (■), and autumn (o)) on the colostrum Brix value (°Bx) in Murciano–Granadina goats.

The effects of season and the number of postpartum milkings on the Brix value (°Bx), recorded using the digital refractometer, are reported in Figure 5, where a statistically significant effect of season was found (*p* < 0.05). The highest Brix values were found in the winter, autumn, and summer samples (17.1, 16.6, and 15.9 °Bx, respectively) compared to spring colostrum (15.4 °Bx); however, summer and spring samples were statistically not different in regard to their Brix value. For milking day, a linear decrease in Brix value (*p* < 0.001) was observed between the first, second, and third days, stabilizing from the third day onwards (21.0, 16.4, 14.2, and 13.4 °Bx for the first, second, third, and fourth milkings, respectively).

### 3.5. Correlation Coefficients

Table 1 presents the Pearson correlations between the chemical components, IgG and LF concentrations, and °Bx of Murciano–Granadina goat colostrum. In general, fat is not correlated with the other components, except for with SNF (r = 0.266, *p* < 0.01); meanwhile, protein colostrum content is negatively correlated with lactose (r = −0.835, *p* < 0.001) and positively correlated with (*p* < 0.001) with SNF (r = 0.983), IgG (r = 0.864), LF (r = 0.705), and °Brix (r = 0.934). Lactose is negatively correlated (*p* < 0.001) with SNF (r = −0.727), IgG (r = −0.815), LF (−0.571), and °Brix (r = −0.812). SNF is positively correlated (*p* < 0.001) with IgG (r = 0.815), LF (r = 0.688), and °Brix (r = 0.904). Finally, IgG is positively correlated (*p* < 0.001) with LF (r = 0.757) and °Brix (r = 0.894). The Brix value was positively correlated with LF concentration (r = 0.716, *p* < 0.001).

## 4. Discussion

The goat colostrum composition in the first few days after birth and in milk production is greatly influenced by many factors, such as breed, diet, stage of lactation, feeding, environment, littler size, udder health, and individuality between goats, among others. Thus, even within a single breed, genetic differences between individual goats can result in variations in colostrum composition, leading to unique compositions for each animal [10,18]. In this experiment, the effects of seasonal variation and postpartum time on the yield and composition of Murciano–Granadina dairy goat colostrum were examined.

Variations in colostrum yield during different stages of lactation and seasons are influenced by a combination of physiological, environmental, and management factors. Regarding the stage of lactation, the mammary gland’s ability to produce colostrum is at its peak right after parturition. As lactation progresses, the composition of milk changes from colostrum to mature milk, typically within the first 3–5 days postpartum, and the yield decreases as colostrum transitions to mature milk. Parity season is one of the main variables responsible for the productivity and quality of colostrum, as climatic conditions (humidity, temperature, and photoperiod) can stress the animal, leading to reduced colostrum yield. Thus, in cows, heat stress affects the metabolism and reduces feed intake, leading to lower colostrum production. Conversely, cold stress can increase energy demands for maintaining body temperature and decrease the energy available for colostrum yield [19,20]. In addition, the season also influences pasture availability and forage quality, as well as water intake, causing variations throughout the year. It is known that significant variations in colostrum yield occur during different seasons and stages of lactation within milking cows [21]. However, dairy goats are different in some respects, and milk production is not as affected by heat stress, as this species is more adapted to high temperatures [22]. The average production values for goats in the first 4 days after kidding in the present study were affected by season and time postpartum, and were consistent with the values and evolution previously described for Murciano–Granadina goats [10,22]. Seasonal differences have been reported in the literature for other breeds [23]. Summer lactations showed higher productivity compared to those in autumn and spring, respectively, while winter kiddings were the least productive. In summer, pasture availability is lower and water consumption increases. However, our trial was carried out in an intensive system where the animals were continuously fed, although they were kept under natural environmental conditions, and in summer, the higher temperature caused increased water intake. Our results agree with those reported by Bermejo et al. [20], who studied the variation in milk production and curve-shape parameters in primiparous Murciano–Granadina goats, showing that late summer lactations tend to result in higher and longer-lasting initial productions.

Seasonal factors such as diet, environmental stress, changes in hormonal status, or exposure to different pathogens can affect the composition of goat colostrum [11,12]. Our study found that most of the chemical components of colostrum were influenced by the seasonal period, although the lactose content was not significantly different in the analyzed seasons. Similar seasonal variations in basic colostrum parameters were obtained in previous studies [11,24]. Regarding fat content, the values for the first milking ranged from 6.32% in summer to 9.19% in autumn, aligning with the results of Ruiz et al. [11] for the Murciano–Granadina breed, as they observed the highest fat content in colostrum during autumn (6.05%). A similar trend was observed for colostrum protein concentration. This result can be explained by the fact that, in these seasons, animals tend to eat more due to the cold weather and also tend to lay down fat for heat conservation [25]; additionally, water consumption decreases in cold seasons, leading to an increase in the SNF and fat contents of milk.

As expected, postpartum time significantly affected most of the chemical parameters of the goat’s milk, except for colostral fat content. Published work indicates highly variable values of colostral fat, which is probably due to the fact that this component is more affected by the goat’s diet than by postpartum time [11,26]. The fat in colostrum is a very important energy concentrate for the newborn.

With respect to the rest of the components, the overall trend during the first four days postpartum was a rapid decrease in colostral proteins and NFS content. Thus, in our study, a higher protein percentage on the first postpartum day was observed. The same increasing trend for lactose and decreasing trend for protein has been reported previously in different goat breeds [2,10,11,27]. Lactose is an alternative major source of glucose [28] and is an energy compound that has a faster assimilation than fat [27], meeting newborn energy needs.

Among the protein components of colostrum, we highlight the high content of immunoglobulins on the first postpartum day, as well as other multifunctional proteins, such as LF, which decreased significantly in the following postpartum days in agreement with other studies [11,29]. As previously described, the passive transfer of immunoglobulins from colostrum has been widely recognized as being essential for neonatal health [3,4,30]. Of all the immunoglobulins present in goat colostrum, IgG is quantitatively the most important, so we focused our analysis on it. Preliminary studies have reported different mean IgG concentrations in colostrum from dairy goats, with values ranging from 28.2 mg/mL in the Murciano–Granadina breed [10] to 72.1 mg/mL in Saanen goat colostrum [31], as well as intermediate values (41.2 and 47.3 mg/mL,) for the Majorera and Alpina breeds, respectively [27,32]. In addition to breed, the level of IgG in colostrum can be affected by other factors, such as age, diet, animal health [33], and lactation time, as we have observed in this research. The highest colostrum concentrations of IgG (30.61 mg/mL) were observed on the first day after birth, but they declined by around 42% within the initial 24 h and up to 87% at 96 h postpartum. This underscores the critical importance of ensuring that the kid receives colostrum from the dam as early as possible.

The lactoferrin levels in goat colostrum over the first 96 h postpartum were influenced by both time and season. The LF concentrations found in this study between the first and fourth postpartum days (means = 413.11 ± 13.85 μg/mL) were higher than those reported by Hiss et al. [9] for goat colostrum, and they also exceeded the range documented by Rachman et al. [6] for several goat breeds during the first 8 days of lactation. Like for IgG, the highest level of LF was observed during the first milking and decreased throughout the first days of lactation by about 29% within the initial 24 h and up to 50% at 96 h postpartum. The decrease in LF concentration during the transition from colostrum to milk followed a similar pattern to postpartum changes in IgG levels; however, the reduction in IgG concentration was more pronounced compared to the decrease in lactoferrin content, which is consistent with the findings of other authors [34]. The levels of lactoferrin in normal milk vary considerably according to species [35,36], breed, milking frequency [6], and diet. According to Mackle et al. [37], the concentration of milk whey proteins is significantly influenced by the energy supplied in the feed ration. Similarly, a study using cow’s milk analyzed lactoferrin contents with varying degrees of access to pasture. They found that cows with unrestricted access to pasture (ad libitum) produced significantly higher amounts of lactoferrin [38]. In contrast to these studies, Navarro et al. [34], in their study with colostrum and sheep milk, concluded that the high variability in lactoferrin concentration throughout lactation, particularly in the first few days, may depend more on the individual than on breed or nutritional factors.

The seasonal effect on LF content in goat colostrum indicated significantly higher values in the spring season. These results are in agreement with those of Brodziak et al. [39], who reported a remarkable seasonal effect on LF concentration during the spring–summer season compared to the autumn–winter season in a large population of samples from several goat breeds. Litwińczuk et al. [40] found a similar seasonal effect on LF content in cow’s milk from Polish local breeds.

On the other hand, LF levels in milk rise quickly during udder infections, such as mastitis, indicating immune response activity [29]. The incidences of mastitis and somatic cell count (SCC) in milk are influenced by season, which may be associated with innate immune functions, including antimicrobial components in the mammary glands, such as LF [41]. However, in our study, we did not find any relationship between LF and SCC. In addition, recently, El-Sharawy et al. [8] observed that LF had immunomodulatory properties, favoring growth in kids treated with it, emphasizing the importance of this protein.

It is noteworthy that, although there was a decrease in the main components analyzed as a function of postpartum day, the magnitude of the decrease was different according to the season, which led to the observed diet x season interactions. Thus, while the protein concentration dropped by almost 20% from the first to the second day in summer, it dropped by over 40% in autumn. In relation to IgG, the reduction from day 1 to day 2 was 68% in autumn, while in summer, it was 46%. A similar situation was observed for lactoferrin, a reduction in which was observed from the first to the second day and ranged from 40% in summer to 15% in spring; from the second day to the third day, its concentration decreased negligibly in winter and by 36% in autumn.

A Brix refractometer is a rapid, accurate, and inexpensive tool that depends on the ability of protein to refract light for predicting the IgG content of colostrum [1,42]. Thus, our findings indicated a strong positive correlation between the °Bx and IgG colostrum content, as well as the contents of other protein components, such as LF. The Brix values in colostrum from the first milking ranged from 19.9 to 22.0 °B, which, according to our ELISA, would correspond to 26.7 and 37.6 mg IgG/mL. However, in colostrum from the second to fourth milkings, there was a significant decrease in Brix values (16.2–13.3 °Bx), following the trends observed in the IgG ELISAs (12.4–3.8 mg IgG/mL). To guarantee successful immunization of newborn kids, IgG colostrum values higher than 20 mg/mL are considered suitable and an appropriate cut-off point for Brix refractometry [13]. In our analysis, this IgG content corresponded to 18 °Bx, similar to the 18–21 °Bx range previously recommended for goat colostrum [13,15,43,44]. Therefore, only the colostrum from the first milking would be adequate for the appropriate immunization of newborns.

## 5. Conclusions

Overall, the results of this research confirm that the chemical components, immunological properties, and lactoferrin content of goat colostrum produced in an intensive system decreased from the first to the fourth milking postpartum. Regarding seasonal effects, lactoferrin was affected by season, being lower in winter colostrum. The highest goat colostrum yields were obtained in summer, with lower fat and protein content; however, immunological properties were not affected. Additionally, this study validates the usefulness of the Brix refractometer for estimating colostral IgG content. Our findings also underscore the critical importance of colostrum feeding within the first postpartum day due to its superior quality, particularly for immunological content and other functional proteins.

## Figures and Tables

**Table 1:** Pearson correlation coefficients between chemical composition, immunoglobulin G (IgG) and lactoferrin content, and Brix value of Murciano–Granadina goat colostrum.

Parameters	Fat	Protein	Lactose	SNF	IgG	Lactoferrin	°Bx
Fat	-	0.221	−0.121	0.266 (**)	0.036	0.099	0.240
Protein		-	−0.835 (***)	0.983 (***)	0.864 (***)	0.705 (***)	0.934 (***)
Lactose			-	−0.727 (***)	−0.815 (***)	−0.571 (***)	−0.812 (***)
SNF				-	0.815 (***)	0.688 (***)	0.904 (***)
IgG					-	0.757 (***)	0.894 (***)
Lactoferrin						-	0.716 (***)
°Bx							-

Fat (%), protein (%), lactose (%), SNF non-fat solids (%), IgG (mg/mL), lactoferrin (mg/L), and °Bx (%). *** *p* < 0.001; ** *p* < 0.01.

## Data Availability

The data presented in this study are available upon request from the corresponding author (F.H.).
